# TRDMT1 participates in the DNA damage repair of granulosa cells in premature ovarian failure

**DOI:** 10.18632/aging.203080

**Published:** 2021-06-08

**Authors:** Chunli Sha, Lu Chen, Li Lin, Taoqiong Li, Hong Wei, Meiling Yang, Wujiang Gao, Dan Zhao, Qi Chen, Yueqin Liu, Xiaofang Chen, Wenlin Xu, Yuefeng Li, Xiaolan Zhu

**Affiliations:** 1 Reproductive Center, The Fourth Affiliated Hospital of Jiangsu University, Zhenjiang 212001, Jiangsu, China; 2 Department of Central Laboratory, The Fourth Affiliated Hospital of Jiangsu University, Zhenjiang 212001, Jiangsu, China; 3 Department of Radiology, The Affiliated Hospital of Jiangsu University, Zhenjiang 212001, Jiangsu, China; 4 International Genome Center, Jiangsu University, Zhenjiang 212013, Jiangsu, China

**Keywords:** premature ovarian failure, ROS, TRDMT1, DNA damage, granulosa cells

## Abstract

The molecular mechanisms underlying premature ovarian failure, which seriously impacts the physical and psychological health of patients, are not fully understood. Here, we present the role of TRDMT1 in reactive oxygen species-induced granulosa cells death, which is considered an important cause of premature ovarian failure. We found that reactive oxygen species were increased in a H_2_O_2_ dose-dependent manner and accompanied by the nuclear shuttling of TRDMT1, increased DNA damage and increased apoptosis of granulosa cells. In addition, reactive oxygen species-induced granulosa cells apoptosis could be prevented by the antioxidant N-acetylcysteine or overexpression of TRDMT1. Furthermore, DNA repair following reactive oxygen species induction was severely impaired/enhanced in TRDMT1 mutants, which exhibited reduced/increased RNA m5C methylation activity. Altogether, our results reveal a novel role of TRDMT1 in the regulation of premature ovarian failure through the repair of reactive oxygen species-triggered DNA damage in granulosa cells and provide an improved understanding of the mechanisms underlying granulosa cells apoptosis, which could potentially be useful for future clinical treatments of premature ovarian failure.

## INTRODUCTION

As a significant cause of female infertility, premature ovarian failure (POF) is characterized by amenorrhea, low estradiol (E2) levels (<20 pg/ml), elevated gonadotropin levels (FSH>20 IU/l), low AMH levels <0.5 ng/ml, and low statin B levels as measured at least twice in a 4-6 week timeframe in women aged under 40 years [1–3]. POF affects up to 1% of infertile women of reproductive age [4], and the known causes of this condition include genetic aberrations, autoimmune ovarian damage, and iatrogenic and environmental factors [5]. However, the underlying mechanisms have not been fully elucidated, and insights into both the disease etiology and targeted intervention are needed.

During the prolonged reproductive lifespan of women, granulosa cells (GCs) connected to oocytes play critical roles in maintaining the follicle reservoir, oocyte growth and follicular development [6, 7]. GCs are essential for follicle development because they support the developing oocyte and its proliferation and the production of sex steroids and disparate growth factors [8]. Increased DNA damage and reduced DNA repair in GCs may contribute to ovarian aging [9, 10]. Chemotherapy drugs that induce DNA damage in GCs and elicit an ovarian DNA repair response exhibit significant toxicity in the reproductive system and have detrimental effects on folliculogenesis, leading to irreversible POF [11]. The mechanism by which cyclophosphamide (CTX) and/or its main metabolite acrolein affect female fertility remains unclear, but this effect is thought to be caused by an overproduction of reactive oxygen species (ROS) [9]. ROS can induce DNA breaks and oxidized bases, and ROS generation has been shown to be associated with the inhibition of mammalian ovarian follicular development and GCs impairment; therefore, ROS generation is responsible for the age-related loss of cellular functions and represents the main cause of aging [12–14]. Evidence suggests that increased intracellular ROS concentrations result in GCs apoptosis in mice [15]. In H_2_O_2_-treated mouse models, oxidative damage has been shown to block GCs development and trigger follicular atresia [16]. Fetal mesenchymal stem cells (fMSCs) significantly decrease oxidative damage, increase oxidative protection, improve antiapoptotic effects, and inhibit apoptotic genes *in vivo* and *in vitro* [17]. CoQ10 administration significantly enhanced the histological morphology and number of atretic follicles in the ovaries of CTX-treated mice by downregulating the ROS levels [18]. In ovarian cancer, the ROS inhibitor antioxidant N-acetylcysteine (NAC) significantly attenuated the induction of DNA damage and the perturbation of proliferation caused by RAD51 depletion [19]. However, the mechanism underlying ROS-induced GCs injury and follicular atresia remains largely unknown.

TRDMT1 is a highly conserved RNA methyltransferase [20] that is believed to participate in the recognition of DNA damage, DNA recombination, and mutation repair [21]. TRDMT1-depleted cells have been found to be sensitive to oxidative stress conditions as determined by the increased production of ROS and susceptibility to DNA damage, resulting in the inhibition of cell proliferation [22]. TRDMT1 is also considered a driver of fruit fly longevity and a modulator of the stress response [23]. Previously, we found that the RNA modification m5C was specifically induced at sites of DNA damage. Furthermore, TRDMT1 was found to be recruited to DNA damage sites and was required for the induction of RNA m5C modification. Importantly, the loss of TRDMT1 compromised homologous recombination (HR) and increased cellular sensitivity to ROS-induced DNA damage, suggesting that TRDMT1-mediated posttranscriptional modifications of RNA can serve as a DNA damage code regulating DNA repair in GCs [24].

The mechanism of action by which ROS induce GCs apoptosis, which plays a vital role in initiating follicular atresia and ultimately contributes to POF, is not well understood. In this study, we present the role of TRDMT1 in ROS-induced GCs apoptosis by clarifying the pathological changes in GCs in POF, which may represent a novel adjuvant therapeutic strategy for POF.

## RESULTS

### Reduced TRDMT1 is associated with decreased ovarian function

**Figure 1:**
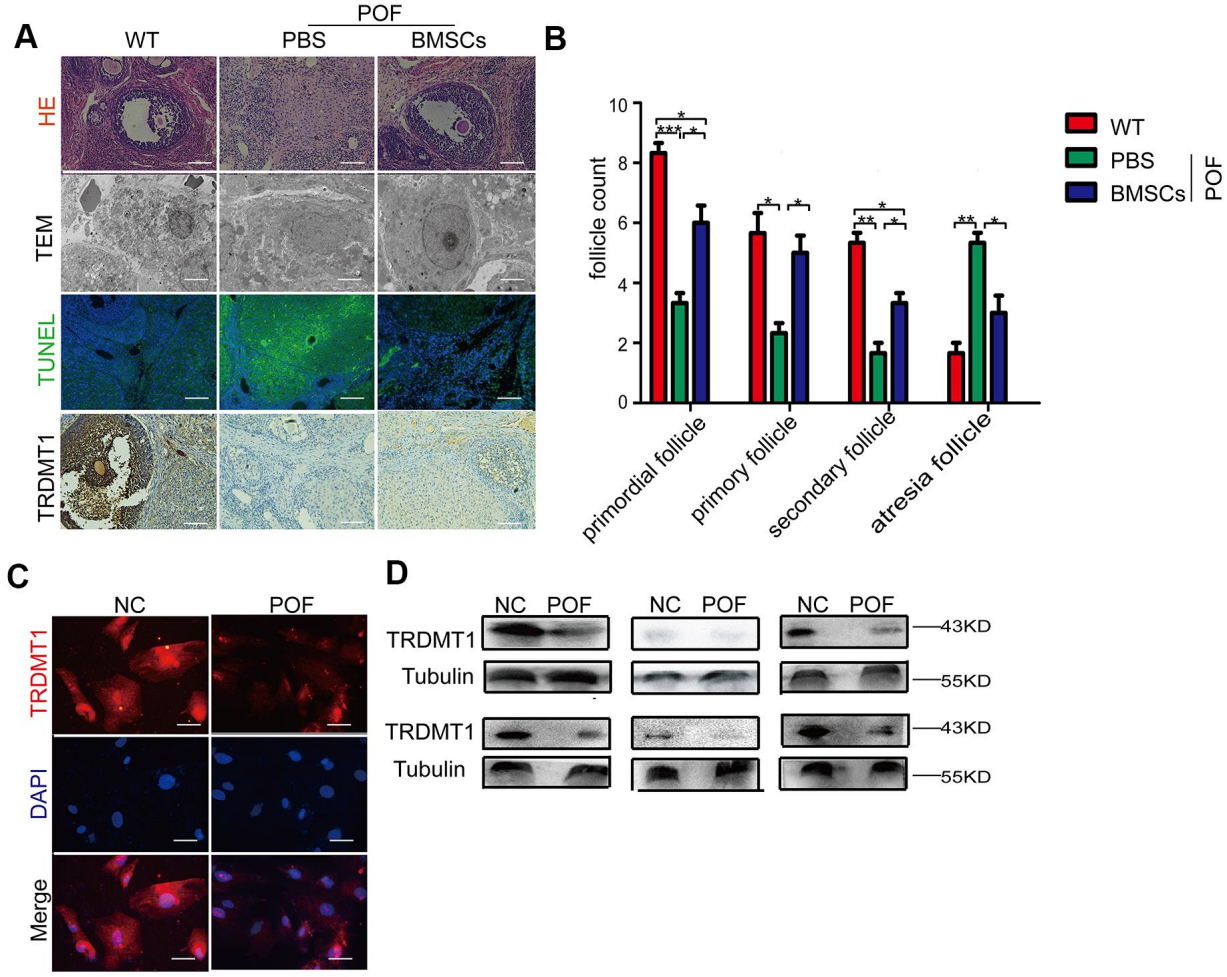
**Reduced TRDMT1 is associated with decreased ovarian function.** (**A**) Histopathological examination of the ovaries of the WT, POF-PBS and POF-BMSCs groups. Scale bar: 100 μm. (**B**) Primordial follicles, primary follicles, secondary follicles, and atretic follicles were observed. (**A**) Transmission electron microscopy analysis of the ovarian structures in each group of mice. Scale bar: 2μm. (**A**) Apoptosis of GCs in ovaries was measured by TUNEL staining. Representative images of terminal deoxynucleotidyl transferase dUTP nick end labeling (TUNEL) staining show apoptotic GCs in each group. The TUNEL-positive apoptotic cells are indicated by green fluorescence. The nuclei (blue) were stained with DAPI. Scale bar: 100μm. (**A**) Representative immunohistochemical images of TRDMT1 in each group. Brown staining represents a positive TRDMT1 signal. Scale bar: 100μm. (**C**) Representative immunofluorescent staining of TRDMT1 in GCs from women with normal ovarian function and from patients with POF. Nuclei were stained with DAPI. Scale bar: 50μm. (**D**) TRDMT1 expression in the serum of healthy people and patients with POF was detected by Western blotting. An anti-Tubulin antibody was used as a loading control. *p<0.05, **P<0.01, and ***P<0.001. Statistical significance was determined using two-tailed t-tests for two groups and ANOVA for multiple comparisons. All values are means ± SD.

To determine whether TRDMT1 is related to a decline in ovarian function, we established a model of POF with CTX followed by bone marrow mesenchymal stem cells (BMSCs) therapy as previously described [25]. The POF rat model was successfully established as shown by a disordered estrus cycle (Supplementary Figure 1A, 1B), increased FSH and LH hormone levels, reduced AMH and E2 values (Supplementary Figure 1D), and decreased ovary weight (Supplementary Figure 1C) and follicular reserve. As expected, the BMSCs injection had a positive therapeutic effect (Figure 1A, 1B). The TEM analysis of the ultrastructure of the GCs is shown in Figure 1A. The GCs in the POF-PBS group exhibited nuclear dissolution, disappearance of the nuclear membrane, chromatin shrinkage, edge aggregation, apoptotic body appearance and mitochondrial vacuolation. In the POF-BMSCs group, the number of organelles decreased, the nuclear membrane of the GCs was gradually repaired, nucleoli appeared, and a small number of mitochondria was present. Unsurprisingly, the ovaries in the POF rat model showed increased tissue apoptosis, which was significantly reduced by the BMSCs treatment (Figure 1A). Interestingly, the expression level of TRDMT1 showed the opposite trend (Figure 1C). These findings suggest that TRDMT1 may be linked to impaired ovarian function. To further confirm the correlation between TRDMT1 and POF, we detected the protein expression level of TRDMT1 in serum and GCs from POF patients undergoing IVF-ET. TRDMT1 in serum and GCs from the POF patients was significantly reduced compared with that from women with normal ovarian function; furthermore, in the POF patients, there was an increase in GCs apoptosis (Figure 1D and Supplementary Figure 1E). The above data show that the expression of TRDMT1 is reduced in ovaries with decreased function, and TRDMT1 reduction is related to the increased apoptosis of GCs.

### TRDMT1 participates in the regulation DNA damage and apoptosis in GCs

**Figure 2:**
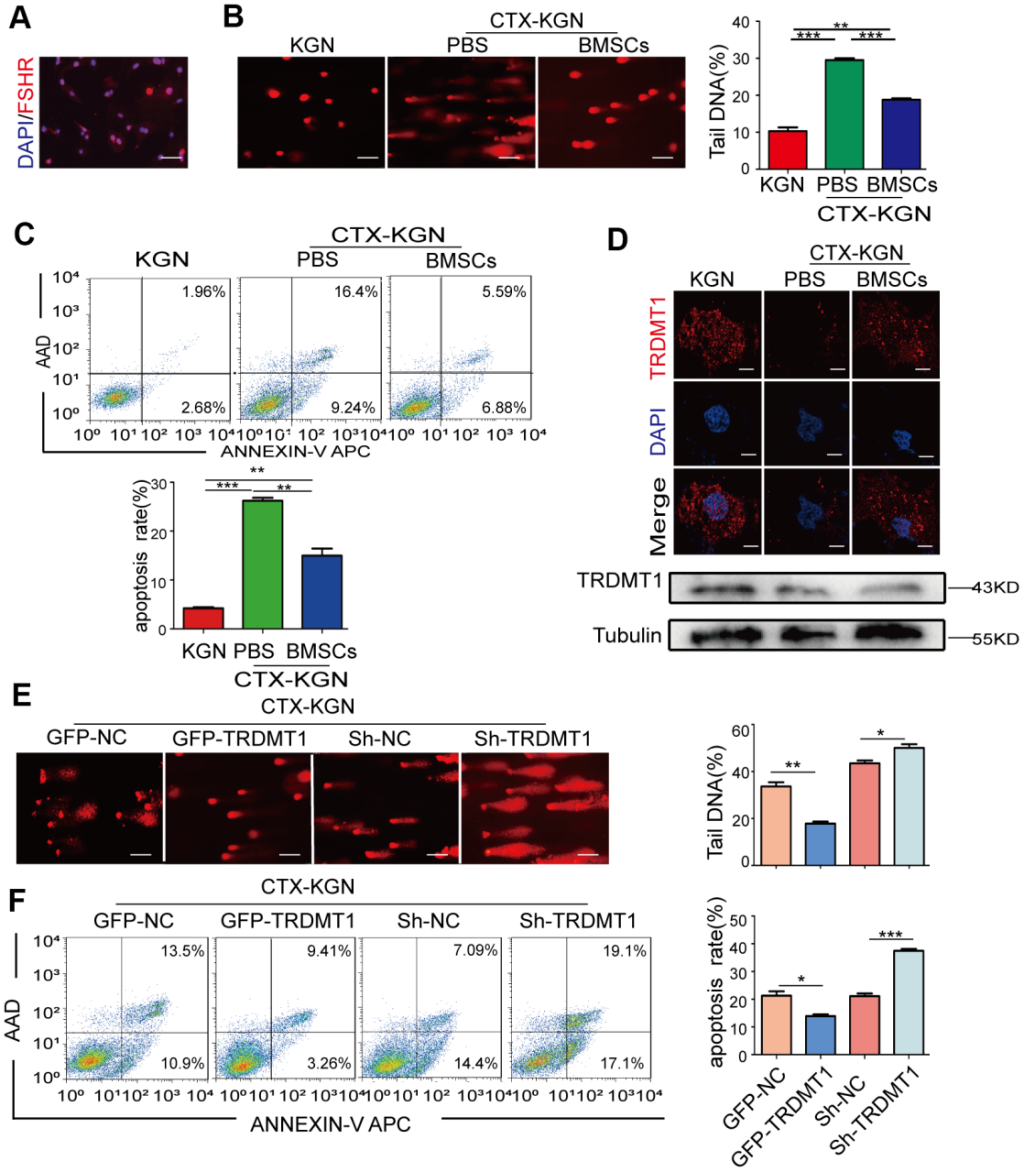
**TRDMT1 is involved in the regulation of GCs DNA damage and apoptosis.** (**A**) Cells with FSHR expression are shown in red. Cell nuclei are stained blue. Scale bar: 100μm. (**B**) DNA damage of KGN, CTX-KGN-PBS and CTX-KGN-BMSCs was detected by comet assay. Scale bar: 100μm. (**C**) Annexin V-APC/AAD staining and flow cytometry analysis of the apoptotic rates of cells in the WT, CTX-KGN-PBS and CTX-KGN-BMSCs groups. Quantification of the apoptosis rates is shown. (**D**) Immunofluorescence staining and Western blotting of the expression levels of TRDMT1 in each group. Scale bar: 10μm. (**E**) DNA damage of CTX-KGN transfected with GFP-NC, GFP-TRDMT1, Sh-RNA and Sh-TRDMT1 was measured by comet assay. Comparison of the tail DNA of cells in the four groups. Scale bar: 100μm. (**F**) Annexin V-APC/AAD staining and flow cytometry analysis of the apoptotic rates of CTX-KGN transfected with GFP-NC, GFP-TRDMT1, Sh-RNA and Sh-TRDMT1. Comparison of the percentage of apoptotic cells in the four groups. *p<0.05, **P<0.01, and ***P<0.001. Statistical significance was determined using two-tailed t-tests for two groups and ANOVA for multiple comparisons. All values are means ± SD.

Given that DNA damage repair in cells and tissues is the main process affecting cell survival and apoptosis [26], we explored whether the occurrence and development of POF is related to GCs DNA damage. First, we extracted and identified GCs from rats by conducting a morphological analysis (Supplementary Figure 2A), immunofluorescence analysis of FSHR, and Cy3 staining (Figure 2A). An *in vitro* POF model (CTX-KGN) was established. Then, we performed a comet electrophoresis experiment to estimate the DNA damage in the KGN cells. The DNA tail length in the KGN cells in the POF group was significantly higher than that in the NC group (P < 0.001) but was significantly decreased after the BMSCs treatment (P<0.05) (Figure 2B and Supplementary Figure 2B). Consistently, a higher percentage of apoptotic cells was found in the POF group. However, apoptosis in the KGN cells was significantly reduced by coculture with BMSCs (Figure 2C). Importantly, the expression of TRDMT1 was significantly downregulated in CTX-KGN but recovered to some extent in CTX-KGN cocultured with BMSCs (Figure 2D and Supplementary Figure 2C). To further determine the relationship between TRDMT1 and DNA damage and apoptosis in KGN cells, we altered the expression level of TRDMT1 by exogenously adding TRDMT1 to CTX-KGN. CTX-KGN transfected with GFP-NC, GFP-TRDMT1, Sh-RNA and Sh-TRDMT1 were confirmed by a Western blot analysis (Supplementary Figure 1E). As expected, the DNA tail length and apoptosis in CTX-KGN were significantly decreased by TRDMT1 expression but increased by TRDMT1 depletion, indicating that TRDMT1 may promote DNA damage repair and decrease apoptosis in KGN cells (Figure 2E, 2F and Supplementary Figure 2D).

### Oxidative stress induces DNA damage and apoptosis in GCs

The most accepted theory of follicle senescence emphasizes the reduced ability of GCs to counteract ROS as a causative factor [27], ultimately resulting in a reduced follicle number, compromised follicle quality and disrupted reproductive endocrinology in the ovary [28, 29]. Thus, we sought to clarify whether ROS could determine GCs’ state or fate, such as apoptosis. The ROS levels in the GCs from the patients with POF were significantly increased compared with those from healthy people (Figure 3A and Supplementary Figure 3A). Consistently, the increased ROS levels were monitored in our POF *in vivo* and *in vitro* models and could be eliminated by BMSCs (Figure 3B, 3C and Supplementary Figure 3B, 3C). To further explore the underlying mechanism, KGN cells were treated with increasing concentrations of ROS from 0μM to 200 μM for 1 h. The total ROS level in the KGN cells was increased in a dose-dependent manner and accompanied by increased DNA damage as evidenced by increased DNA fragmentation and the delayed disappearance of γ-H_2_AX foci, which is considered a reliable molecular marker of DNA damage and aging (Figure 3D, 3E, 3G). Furthermore, the number of apoptotic KGN cells was increased with increased ROS (Figure 3F), suggesting that oxidative DNA damage may be an important cause of KGN cell apoptosis in the POF model and that ROS-promoted DNA damage and apoptosis in KGN cells may lead to the occurrence of POF. Interestingly, normally, most TRDMT1 was located in the cytoplasm of the KGN cells; however, TRDMT1 was transferred to the nucleus after the H_2_O_2_ treatment (Figure 3H), indicating that TRDMT1 may function in the nucleus and is involved in the repair of oxidative DNA damage in KGN cells. To further detect whether TRDMT1 nuclear-localization have beneficial function in DNA repair, we transfected TRDMT1 mutants into the cells, and observed its cell localization after H_2_O_2_ treatment. Interestingly, nuclear-localization in TRDMT1^WT^ and TRDMT1^E63K^ expressing cells was significantly higher than that of TRDMT1^G155V^ expression (Figure 3H). These results indicate that TRDMT1 mutants affect their subcellular localization and TRDMT1 nuclear-localization have beneficial function in DNA repair.

**Figure 3:**
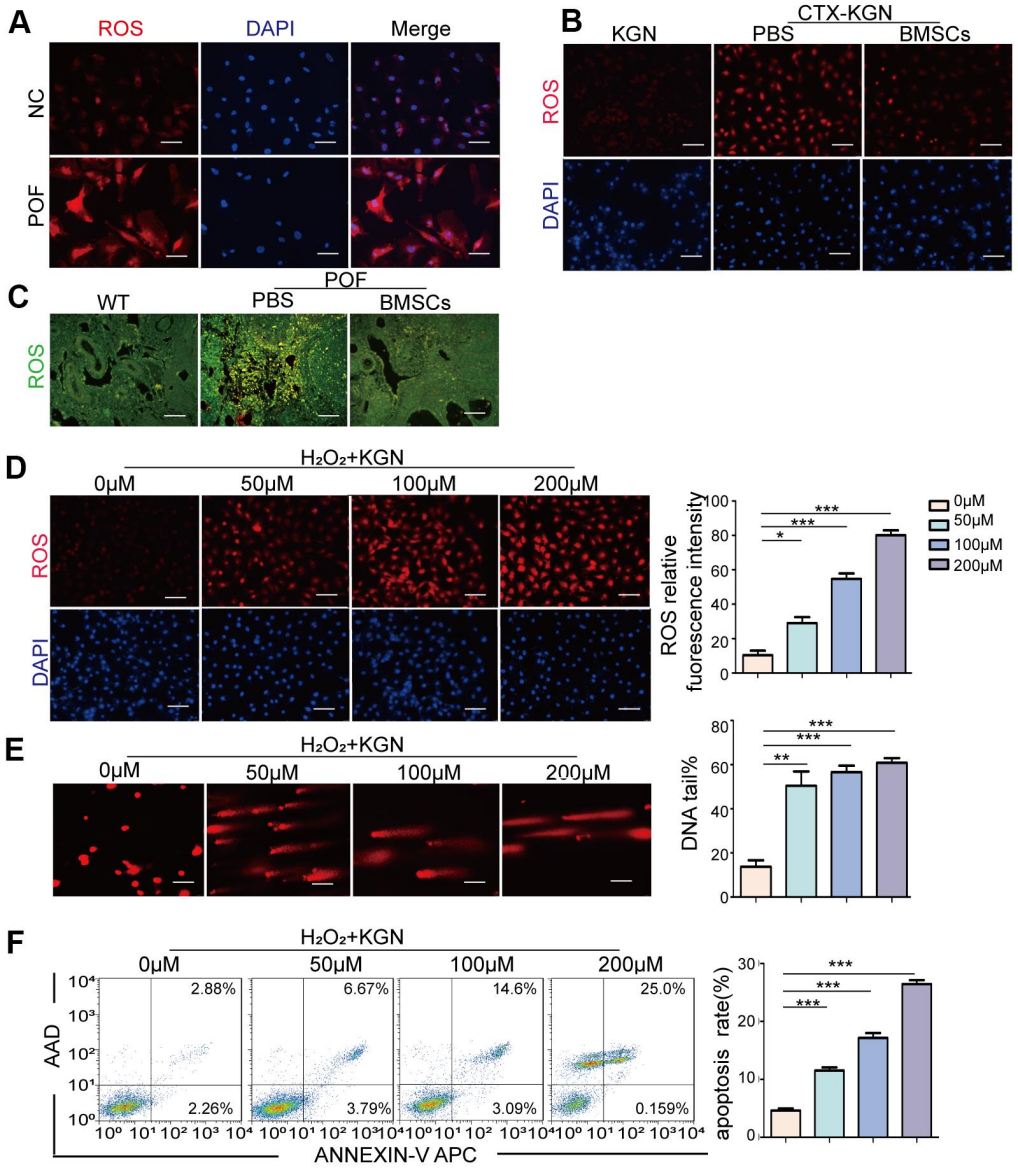
**Oxidative stress induces DNA damage and apoptosis in GCs.** (**A**) ROS of GCs of women with normal ovarian function and POF patients was assessed via DHE probes. Scale bar: 100μm. (**B**) ROS in the KGN, CTX-KGN-PBS and CTX-KGN-BMSCs groups were assessed using DHE probes. Scale bar: 100μm. (**C**) ROS in the ovaries of the WT, POF-PBS and POF-BMSCs groups were assessed using DCFH-DA probes. Scale bar: 100μm. (**D**) Cells were treated with 0 μM to 200 μM H_2_O_2_ for 1 hour. Total production of ROS was assessed using DHE probes. Scale bar: 100μm. (**E**) The DNA damage of cells treated with H_2_O_2_ was measured via comet assay. Scale bar: 100μm. Comparison of the tail of DNA between the groups. (**F**) Apoptosis of H_2_O_2_+KGN was assessed using Annexin V staining. Comparison of apoptotic rates between the groups.

**Figure 3:**
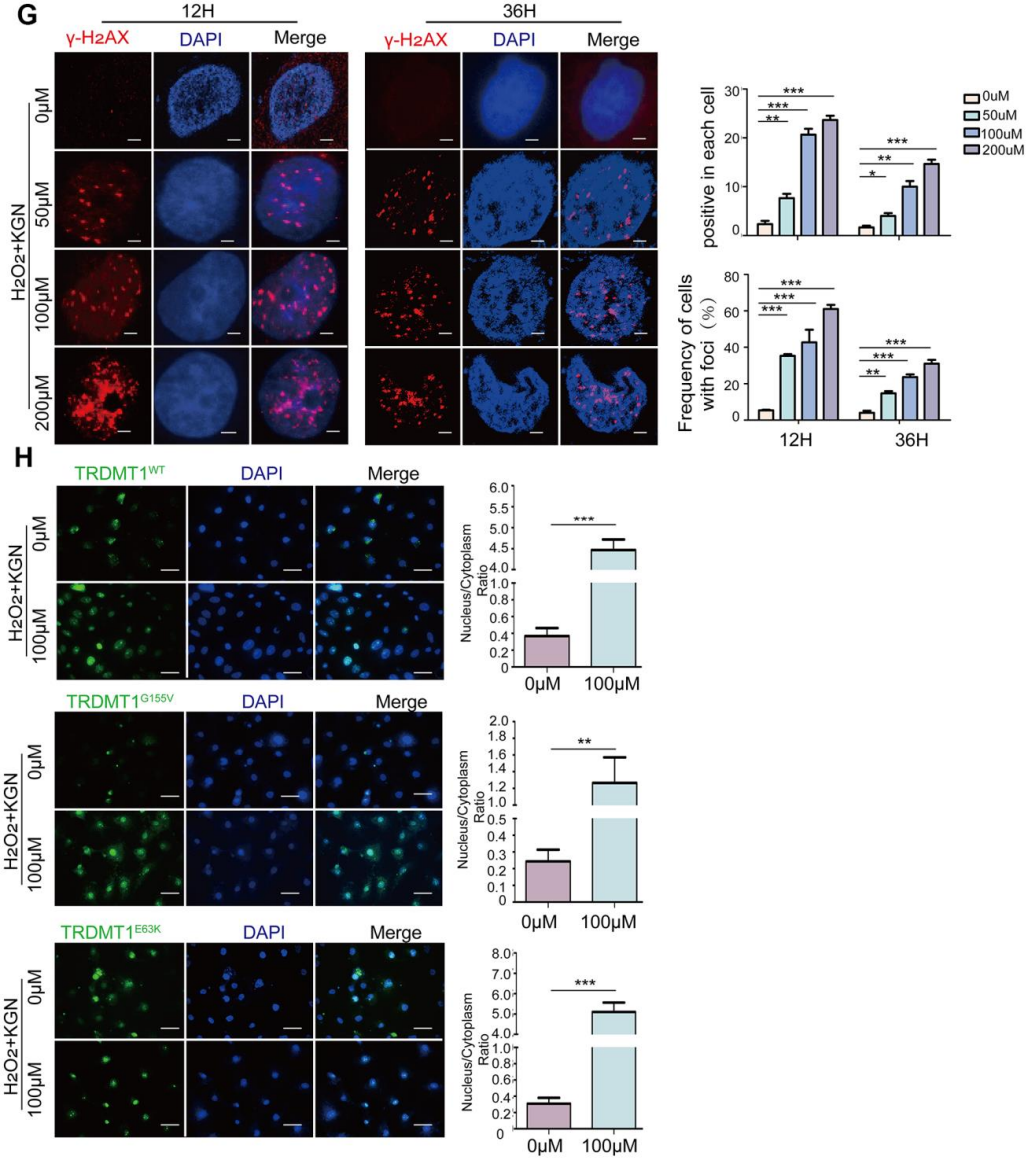
**Oxidative stress induces DNA damage and apoptosis in GCs.** (**G**) H_2_O_2_+KGN were harvested at the indicated time points for γ-H_2_AX staining. Positive cells in each group and the frequency of cells with foci were counted. Scale bar: 10μm. (**H**) KGN cells transfected with GFP-TRDMT1 (WT, G155V, and E63K mutant) were stimulated with H_2_O_2_, and their cell localization was observed. Scale bar: 50μm.Comparison of the nucleus/cytoplasm ratio between the two groups. *P<0.05, **p<0.01, and ***p<0.001. Statistical significance was determined using two-tailed t-tests for two groups and ANOVA for multiple comparisons. All values are means ± SD.

### TRDMT1 mediates oxidative DNA damage repair in GCs

**Figure 4:**
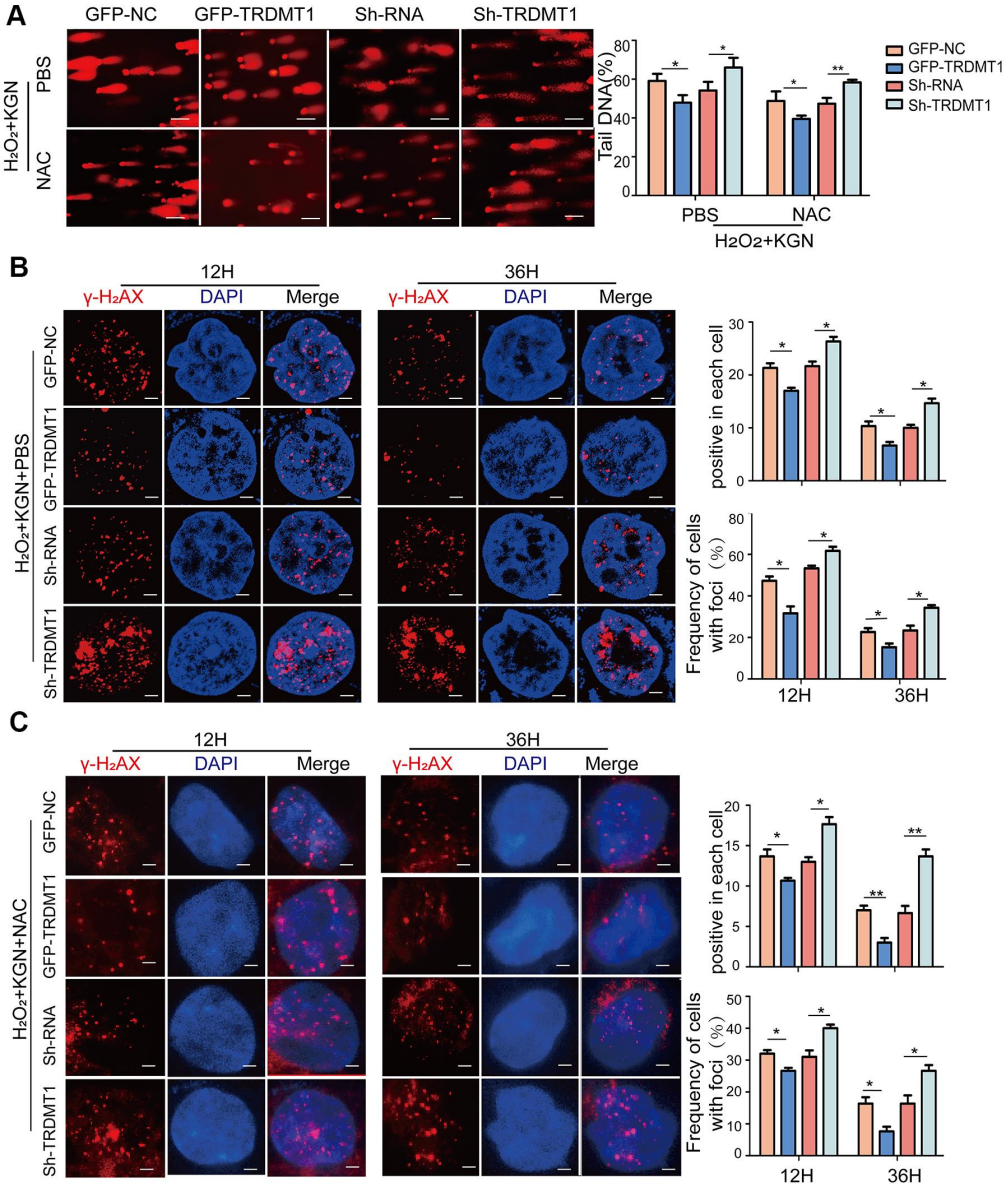
**TRDMT1 mediates GCs oxidative DNA damage repair.** (**A**) The DNA damage of H_2_O_2_+KGN+PBS and H_2_O_2_+KGN+NAC transfected with GFP-NC, GFP-TRDMT1, Sh-RNA and Sh-TRDMT1 was measured via comet assay. Scale bar: 100μm. Comparison of the tail of DNA between the groups. (**B**) H_2_O_2_+KGN+PBS transfected with GFP-NC, GFP-TRDMT1, Sh-RNA and Sh-TRDMT1 were harvested at the indicated time points for γ-H_2_AX staining. Positive cells in each cell and the frequency of cells with foci were counted. Scale bar: 10μm. (**C**) H_2_O_2_+KGN+NAC transfected with GFP-NC, GFP-TRDMT1, Sh-RNA and Sh-TRDMT1 were harvested at the indicated time points for γ-H_2_AX staining. Positive cells in each group and the frequency of cells with foci were counted. Scale bar: 10μm.*P<0.05, **p<0.01, and ***p<0.001. Statistical significance was determined using two-tailed t-tests for two groups and ANOVA for multiple comparisons. All values are the means ± SD.

TRDMT1 was found to be related to ROS levels and DNA damage in both the *in vivo* and *in vitro* models of POF, and ROS is an important trigger of DNA damage. We aimed to explore whether TRDMT1-mediated DNA damage regulation is related to ROS. Therefore, we overexpressed TRDMT1 in KGN cells or knocked down TRDMT1 using H_2_O_2_. As shown in Figure 4A, following the H_2_O_2_ treatment_,_ the DNA tail in the KGN cells was shortened with the TRDMT1 overexpression but was obviously elongated after the TRDMT1 downregulation compared with that in the control group; these data suggest that TRDMT1-depleted cells are genomically unstable and more prone to oxidative DNA damage than normal cells. Consistently, the TRDMT1 inhibition delayed the clearance of γ-H_2_AX foci (Figure 4B and Supplementary Figure 4B). Then, we inhibited the ROS production of H_2_O_2_+KGN by treatment with NAC, and DNA damage in the KGN cells was clearly reduced; furthermore, the DNA damage repair effect mediated by TRDMT1 was significantly weakened as evidenced by the delayed disappearance of γ-H_2_AX foci (Figure 4A, 4C and Supplementary Figure 4A, 4C).

### Oxidative DNA damage repair promoted by TRDMT1 is related to RNA m5C methylation

**Figure 5:**
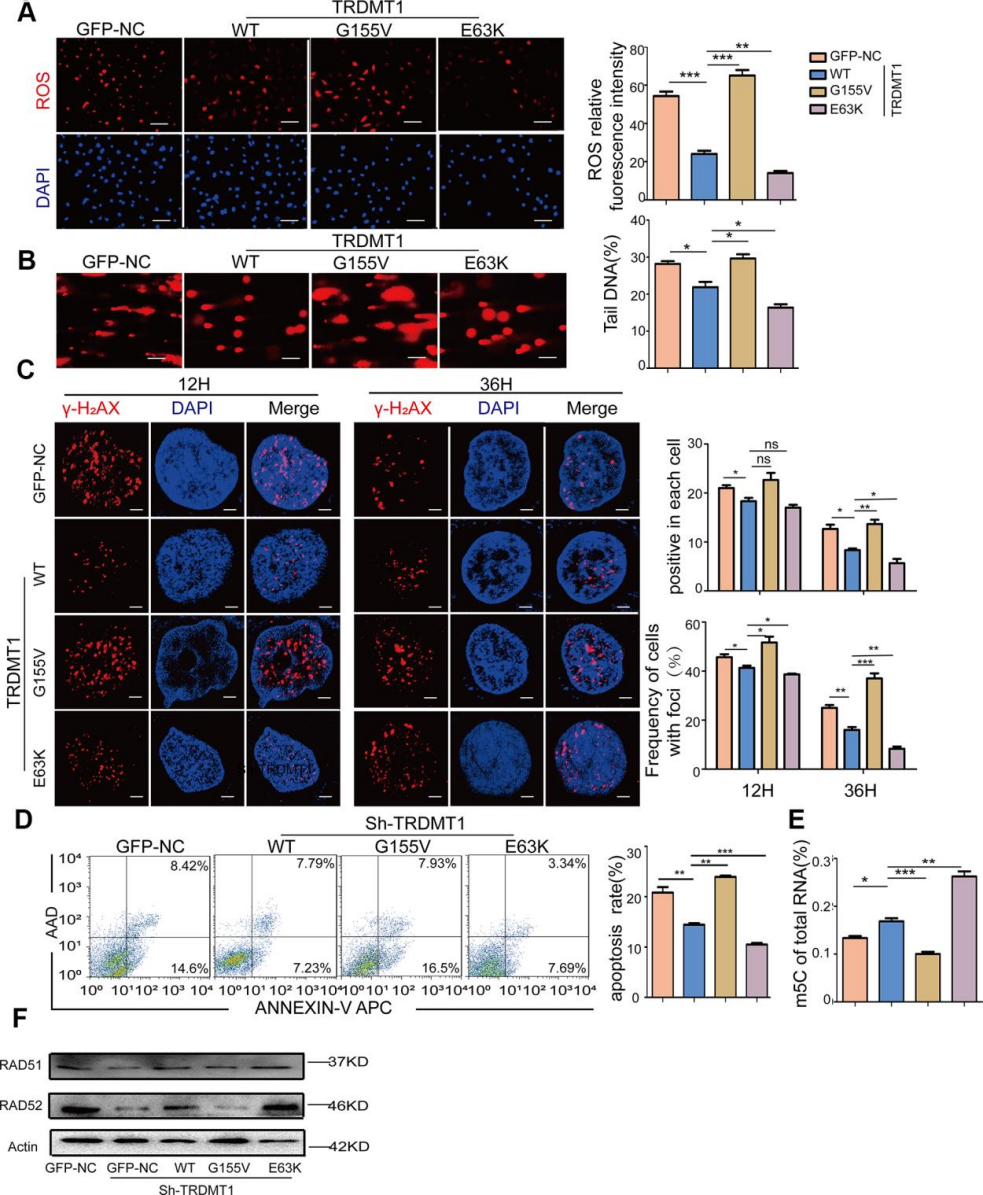
**The oxidative DNA damage repair promoted by TRDMT1 is related to RNA m5C methylation.** (**A**) ROS in CTX-KGN were detected by DHE probe after transfection with GFP-NC or GFP-TRDMT1 (WT, G155V, and E63K mutant). Scale bar: 100 μm. (**B**) The DNA damage of CTX-KGN transfected with GFP-NC or GFP-TRDMT1 (WT, G155V, and E63K mutant) was measured via comet assay. Scale bar: 100μm (**C**) CTX-KGN transfected with GFP-NC and GFP-TRDMT1 (WT, G155V, and E63K mutant) were stained for γ-H_2_AX at the indicated time points. Scale bar: 10μm. Positive cells in each group and the frequency of cells with foci were counted. (**D**) Apoptosis of CTX-KGN transfected with GFP-NC or GFP-TRDMT1 (WT, G155V, E63K mutant) was assessed using Annexin V staining. Comparison of apoptotic rates between the groups. (**E**) The percentage of m5C in total RNA from CTX-KGN after transfection with GFP-NC or GFP-TRDMT1 (WT, G155V, and E63K mutant) was detected via ELISA-based assays. (**F**) The expression of RAD51 and RAD52 were detected after we stably expressed TRDMT^WT^ and the mutants (TRDMT1^G155V/E63K^) in TRDMT1 KD CTX-KGN. *P<0.05, **p<0.01, and ***p<0.001. Statistical significance was determined using two-tailed t-tests for two groups and ANOVA for multiple comparisons. All values are means ± SD.

Our previous study established that ROS induced not only DNA damage but also m5C modifications of RNA. The RNA methyltransferase TRDMT1 is responsible for ROS-induced m5C formation and is critical for the repair of DNA double-strand breaks (DSBs) [24]. Therefore, we sought to determine the link between the TRDMT1-regulated DNA damage response and its m5C methylation activity in KGN cells. Studies have reported that the G155V mutation leads to a considerable reduction in the tRNA methylation activity of TRDMT1 *in vitro*, causing it to be almost inactive, whereas the E63K mutation significantly increased its methylation activity [30]. Our results were consistent with those found in the literature (Figure 5E). Then, we stably expressed GFP-tagged wild-type TRDMT1 (TRDMT^WT^) and mutants (TRDMT1^G155V/E63K^) in TRDMT1 KD CTX-KGN. Notably, in contrast to TRDMT1^WT^, ROS was reduced with TRDMT1^E63K^ expression but enhanced with TRDMT1^G155V^ expression, while the efficiency of DNA damage repair showed the opposite trend, revealing the same trend as the overall levels of m5C RNA methylation (Figure 5A, 5B and Supplementary Figure 5A). Collectively, the oxidative DNA damage repair efficiency of TRDMT1 is related to its RNA m5C methylation activity. TRDMT1 inhibition delayed the clearance of γ-H_2_AX foci (Figure 5C and Supplementary Figure 5B). Apoptosis in CTX-KGN transfected with TRDMT1^G155V^ was more abundant than that in those transfected with TRDMT1^WT^ and TRDMT1^E63K^ (Figure 5D). It was reported that RAD51 functions with RAD52 and other proteins to effect strand exchange during homologous recombination (HR) [31]. To investigate whether TRDMT1 could regulate DNA damage repair directly, RAD51 and RAD52 were detected. As showed, RAD51 and RAD52 was decreased in TRDMT1 KD KGN cells while increased in TRDMT^WT^ and the TRDMT1^E63K^ expressing cells after CTX treatment. (Figure 5F and Supplementary Figure 5C). These results suggested that TRDMT1 may promote DNA damage repair.

## DISCUSSION

**Figure 6:**
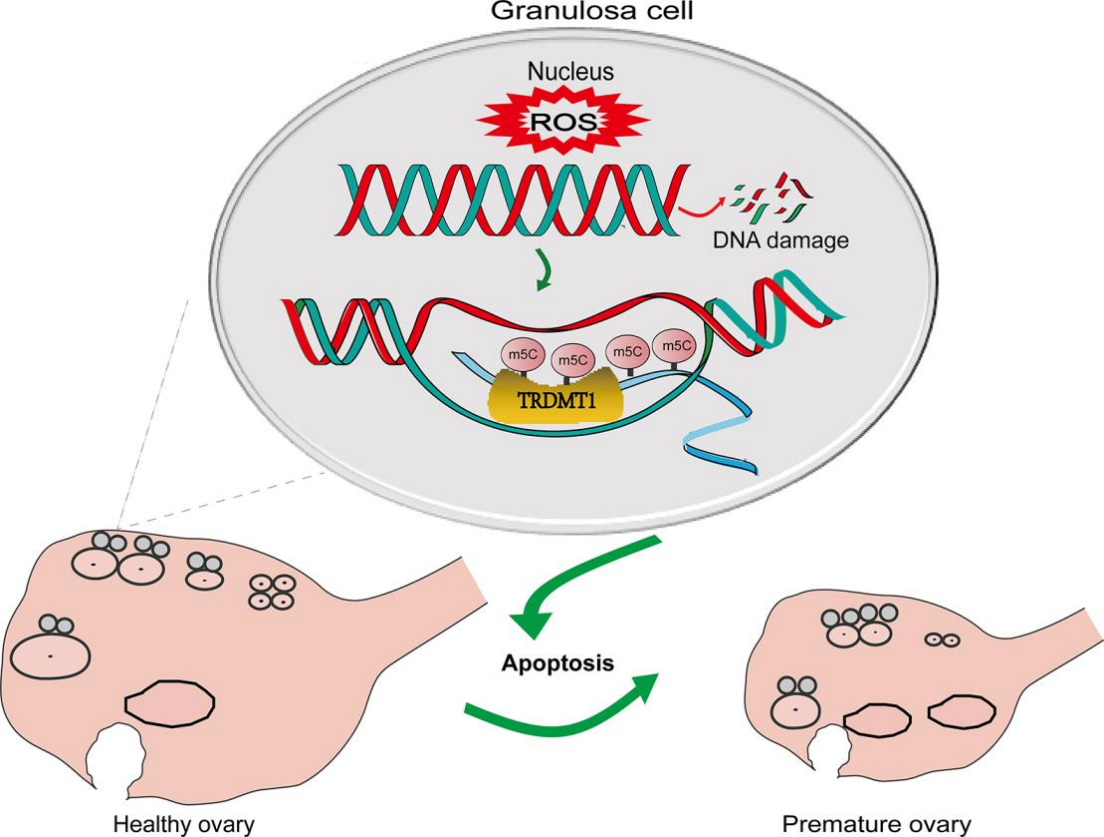
**A schematic diagram showing the possible mechanism of TRDMT1 on GCs.** CTX induced oxidative stress by generating ROS and thereby GCs apoptosis via DNA damage. The increased level of ROS leads to DNA damage. The increase in DNA damage promotes the apoptosis of GCs, thus promoting POF. TRDMT1 is important for DNA damage repair efficiency and survival. TRDMT1 participated in the DNA damage repair of GCs through methylation. m5C mRNA methylation is enriched at transcriptionally active sites with DNA damage. In short, oxidative damage to DNA induced the apoptosis of GCs and promoted the development of POF. TRDMT1 promoted DNA damage repair by regulating the methylation level, which reduced the apoptosis of GCs and inhibited the occurrence of POF. The regulation of oxidative DNA damage repair mediated by TRDMT1 was significantly correlated with its methylation activity.

Apoptosis in GCs is the cellular mechanism responsible for follicular atresia in mammals, which is the main process responsible for the loss of follicles and oocytes from the ovary and the root cause of ovarian aging [32, 33]. DNA lesions that escape DNA repair can induce replication stress and genomic breaks that induce senescence and apoptosis [34] (Figure 6). Icariin exerts a protective effect against D-galactose-induced POF by promoting DNA damage repair [35]. In our POF rat model, increased tissue apoptosis was detected.

Moreover, a higher percentage of apoptotic cells was found in CTX-KGN, which is considered an *in vitro* cell model of POF, suggesting that the increase in KGN cell apoptosis that may be caused by enhanced DNA damage contributes to ovarian aging.

The increased production of toxic metabolites, such as ROS and reactive nitrogen species, and external oxidant agents play an important role in the process of ovarian senescence and the genesis of ovarian pathologies [36]. Levels of ROS higher than physiological levels trigger GCs apoptosis, reduce the transfer of nutrients and survival factors to oocytes, thereby leading to apoptosis, and play a vital role in initiating follicular atresia [37, 38]. ROS can induce DNA damage and DNA fragmentation, which are important and irreversible events in apoptosis [39, 40]. In germ cells, ROS lead to an increase in apoptosis following DNA damage compared to the level observed under physiological conditions [41]. We demonstrated that the ROS levels were significantly elevated in the POF model. H_2_O_2_-mediated oxidative DNA damage increased GCs apoptosis. These results indicate that ROS-promoted cell apoptosis may be a key factor in the progression of POF.

TRDMT1 interacts with proteins involved in RNA processing and is a component of RNA processing bodies and stress granules [42, 43]. Under oxidative stress conditions, the upregulation of TRDMT1 is a part of an adaptive response that protects RNA from degradation [44]. The role of TRDMT1 in stress granules could represent a primitive cellular defense mechanism against viral infection [42]. HP-treated TRDMT1-silenced fibroblasts were more prone to apoptosis than control cells. TRDMT1 may be considered a pro-survival signal during exposure to stress conditions [22]. In our study, TRDMT1 was found to be decreased, and there were elevated levels of ROS in serum and GCs isolated from patients with POF and ovarian tissue from the POF model. TRDMT1 enhanced the repair of oxidative DNA damage, whereas TRDMT1-silenced GCs were more vulnerable to H_2_O_2_-mediated oxidative DNA damage, resulting in increased cell apoptosis. We also found that TRDMT1 nuclear-localization in WT and E63K expression cells was significantly higher than that of G155V expression cells after H_2_O_2_ damage. Besides, in our previous study, in TRDMT1 KO cells, only TRDMT1 tagged with a nuclear localization signal (NLS-TRDMT1) was localized to TA-KR sites. In contrast to NLS-TRDMT1, TRDMT1 tagged with a nuclear export signal (NES-TRDMT1) (mutate the NLS of TRDMT1) could not restore m5C formation and γ-H2AX clearance at the locus marked by TA-KR, nor suppress IR sensitivity. These results suggest that TRDMT1 nuclear-localization is a necessary condition for its beneficial function in DNA repair and TRDMT1 mutants affect their subcellular localization.

We previously demonstrated that the activity of TRDMT1 is required for the efficient repair of ROS-induced DNA double-strand breaks (DSBs). TRDMT1 promoted mRNA m5C modification and DNA repair in the nucleus and recruitment of HR proteins to transcribed damage sites [24]. TRDMT1 knockdown reduced mRNA methylation and altered the patterns of cell proliferation and migration in HEK293 cells through a novel regulatory mechanism [45]. As a part of the regulatory loop of the metabolic pathways directing RNA methylation after nanodiamond treatment, TRDMT1 may contribute to RNA stabilization and confer stress resistance [46]. In the present study, the oxidative DNA damage repair efficiency in the TRDMT1-silenced GCs was increased by TRDMT^WT^ expression and further enhanced by TRDMT1^E63K^ but was impaired by TRDMT1^G155V^ transfection, indicating that the regulation of oxidative DNA damage repair mediated by TRDMT1 was significantly correlated with its methylation activity. It was reported that RAD51 functions with RAD52 and other proteins to effect strand exchange during HR. In mammalian cells RAD51 and RAD52 overexpression increased the frequency of spontaneous HR [31]. RAD51 and RAD52 was decreased in TRDMT1 KD KGN cells while increased in TRDMT^WT^ and the TRDMT1^E63K^ expressing cells after CTX treatment. These results suggest that TRDMT1 can promote DNA damage repair.

## CONCLUSIONS

In summary, our findings imply that TRDMT1 could be beneficial for the preservation of ovarian follicles after chemotherapy-induced ovarian failure through DNA damage repair and that the regulation of oxidative DNA damage repair mediated by TRDMT1 was significantly correlated with its methylation activity. This study not only sheds new light on the role of TRDMT1 in ovarian physiology and pathology but also extends our understanding of potential genes associated with POF in human patients. However, the complex functions of TRDMT1 in the regulation of POF need to be further explored and verified, and how to efficiently apply this approach in the diagnosis of POF still needs to be investigated.

## MATERIALS AND METHODS

### Patient cohort and collection of GCs

The serum samples and GCs used in this study were collected from healthy volunteers and patients with POF who were registered with the Reproductive Medical Center of the Fourth Affiliated Hospital of Jiangsu University. Written informed consent was obtained from each participant in advance, and the research protocol was approved by Jiangsu University Ethics Committee.

### Cell culture and transfection

The human granulosa-like tumor cell line KGN was obtained from Shanghai Ji he Biotechnology Co., LTD, China and maintained in Dulbecco’s modified Eagle’s medium (DMEM)/HamF-12 growth medium (HyClone) with 10% fetal bovine serum and 1% penicillin-streptomycin added. The cells were authenticated by a short tandem repeat assay (STR). GFP-NC and GFP-TRDMT1 were transfected into KGN cells using Lipofectamine 2000 reagent (Invitrogen, USA) following the manufacturer’s instructions.

### Establishment and identification of the POF model *in vivo*

Five-week-old SD female rats were intraperitoneally injected with 50 mg/kg CTX (Baxter Oncology Gmbh, Germany) on the first day and then 8 mg/kg/d CTX consecutively for 14 days as previously described [25]. Vaginal cells were collected using a sterile cotton swab, smeared on glass microscope slides, and stained with crystal violet. The estrous cycle of the SD rats was distinguished according to the cell type.

### Induction of GCs apoptosis *in vitro* and coculture of GCs and BMSCs

CTX was added to the KGN cell culture medium at 50 ng/ml for 24 h to induce apoptosis *in vitro*. The steps of the coculture of KGN and BMSCs were performed as described in our previous study [25]. CTX-damaged KGN cells (CTX-KGN) and H_2_O_2_-damaged KGN cells (H_2_O_2_+KGN) were plated on 6-well plates and transfected using Lipofectamine 2000 according to the manufacturer’s instructions. After 48 h, the cells were collected for further study.

### Hematoxylin and eosin (H&E) staining

Histological observation of ovaries and follicle cell counting. For the histological analysis, the ovaries were fixed in 4% paraformaldehyde (PFA) (Solarbio, CHINA) overnight and embedded in paraffin. The ovarian tissues were cut into sections at 5 μm thickness, mounted on glass slides and stained with H&E. Ovarian primordial, primary, secondary, antral and atretic follicles were counted.

### ELISA

The serum hormones were measured by enzyme-linked immunosorbent assay kits (ImmunoWay, USA). The blood samples were collected during the diestrus period and stored at room temperature for 1 h, followed by centrifugation at 3220 g for 15 min for the serum harvesting. The serum FSH, LH, AMH and E_2_ levels were measured according to the manufacturer’s instructions.

### Tissue transmission electron microscopy

The tissue used for the transmission electron microscopy (TEM) was prepared by fixation in 2.5% glutaraldehyde and resin embedded, and ultrathin sections were examined under a transmission electron microscope.

### TUNEL assay

Terminal deoxynucleotidyl transferase biotin-dUTP nick end labeling (TUNEL) staining was used to assay cellular apoptosis in the ovaries. The apoptotic cells were detected using an *in situ* cell death detection kit, Fluorescin (Beyotime, CHINA). The sections were observed under a fluorescence microscope.

### Immunohistochemistry

The ovaries were fixed in 4% PFA solution and embedded in paraffin. The sections of ovarian tissue were 4 μm thick, dewaxed in gradient alcohol and xylene, and incubated overnight with a TRDMT1 (1:200, mouse monoclonal, Santa Cruz Biotechnology) antibody at 4° C in a humid environment. Then, the sections were incubated with the secondary antibody (mouse monoclonal, Biosharp, CHINA) at room temperature for 2 h.

### Immunofluorescence

The cells used for the immunofluorescence observation were fixed in 4% PFA for 15 min at room temperature and further treated with 0.2% Triton (Solarbio, CHINA) for 10 min. Then, the cells were blocked by 5% BSA (Solarbio, CHINA) for 30 min at room temperature. Primary antibodies against TRDMT1 (1:100, mouse monoclonal, Santa Cruz Biotechnology, USA) were diluted in PBS and incubated with the cells overnight at 4° C. Then, the samples were washed 3 times with TBST. Then, the cells were incubated with secondary antibodies (Biosharp, CHINA) for 1 h at room temperature, followed by washing with PBST 3 times. The incubation with 1:1000 DAPI (Beyotime, CHINA) for 10 min at room temperature was optional. Finally, the labeling was visualized under a fluorescence microscope.

### Western blot analysis

The Western blot analysis of the proteins in the cells and ovarian tissues was performed using sodium dodecyl sulphate-polyacrylamide gel electrophoresis, and the proteins were transferred onto a polyvinylidene fluoride membrane (Bio-Rad Laboratories, Bio Oxford, USA). After blocking, the membrane was incubated with primary antibodies against TRDMT1 and Tubulin (1:1000, mouse monoclonal, Boster, CHINA) combined with a horseradish peroxidase secondary antibody (1:5000, mouse monoclonal, Biosharp, CHINA).

### Comet electrophoresis

The DNA damage in the KGN cells was measured using comet assay kits (Keygen, CHINA). Briefly, 1x10^6^ isolated cells were mixed with 100 μl of low melting point agarose, of which 100 μl were layered on normal agarose slides and allowed to solidify under coverslips at 4° C. After 10 min, the coverslips were removed and placed in lysis buffer for 2 h at 4° C. Then, the slides were placed in electrophoresis solution (300 mM NaOH, 1 mM EDTA) for a 50-min incubation period, followed by 30 min electrophoresis (25 V). The slides underwent neutralization, followed by staining using PI. Then, 50 random cells on each slide were counted under an Olympus epifluorescence microscope. Casp software was used to analyze the DNA tail.

### Estimation of cell apoptosis

After the treatment with CTX or H_2_O_2_, cell apoptosis was determined by flow cytometry (BD Biosciences, USA) using an Annexin V-fluorescein isothiocyanate (APC)/propidium iodide (AAD) kit (Keygen, CHINA) according to the manufacturer’s protocol. The cells were incubated in the dark at room temperature for 10 min. The data were immediately analyzed using an FC500 MCL machine. The fluorescence intensity of the cells was detected by flow cytometry within 1 h. The data were analyzed by FlowJo software (version Win64–10.4.0).

### ROS detection

The redox-sensitive cell-permeable fluorophore DHE (Keygen, CHINA) was used to evaluate the *in situ* production of superoxide. DHE (50μM) was applied to the cells, incubated in a light-protected humidified chamber at 37° C for 1 h, and washed with PBS. Ovary tissues from different groups were stained with 10 μM H2DCFDA fluorescent probe (6-carboxy- 2′,7′-dichlorodihydrofluorescein diacetate) (Solarbio, CHINA) for 30 min at 38° C in the dark. Then, the samples were washed once with PBS, and the images were captured immediately under a fluorescence microscope. ImageJ 1.44p software was used to analyze the fluorescence intensity.

### γ-H_2_AX detection

Primary antibodies against γ-H_2_AX (1:100, mouse monoclonal, EMD Millipore Crop, USA) were diluted in PBS and incubated with the cells overnight at 4° C. The immunofluorescence steps were identical to those described above.

### RNA methylation

ELISA-based assays using a Methyl Flash 5-mC RNA Methylation ELISA Easy Kit (EpiGentek, Farmingdale, USA) were used to measure the levels of 5 methylcytosine (m5C) in the RNA according to the instructions.

### Statistical analysis

The experimental data presented in this paper were based on three parallel experiments and are presented as the mean ± SD. GraphPad Prism software (version 7.0 for Windows) was used to assess the statistical significance. The differences between two groups were analyzed using two-tailed t-tests. One-way analysis of variance (ANOVA) was used for the multiple group comparisons. Bars with different letters indicate statistical significance. P<0.05 was considered statistically significant in this study.

### Ethics statement

The study protocol was approved by the Ethics Committee and Experimental Animal Ethics Committee of Jiangsu University. Written informed consent was obtained from all participants in the study.

### Editorial note

This corresponding author has a verified history of publications using a personal e-mail address for correspondence.

## Supplementary Material

Supplementary Figures
