# Supplementary material for: TRDMT1 participates in the DNA damage repair of granulosa cells in premature ovarian failure
Source: Aging (Albany NY). 2021 Jun 8;13(11):15193–213. doi: 10.18632/aging.203080 (PMC8221345; doi:10.18632/aging.203080)
Supplement: Supplementary Figures [file aging-13-203080-s001.pdf]

SUPPLEMENTARY FIGURES

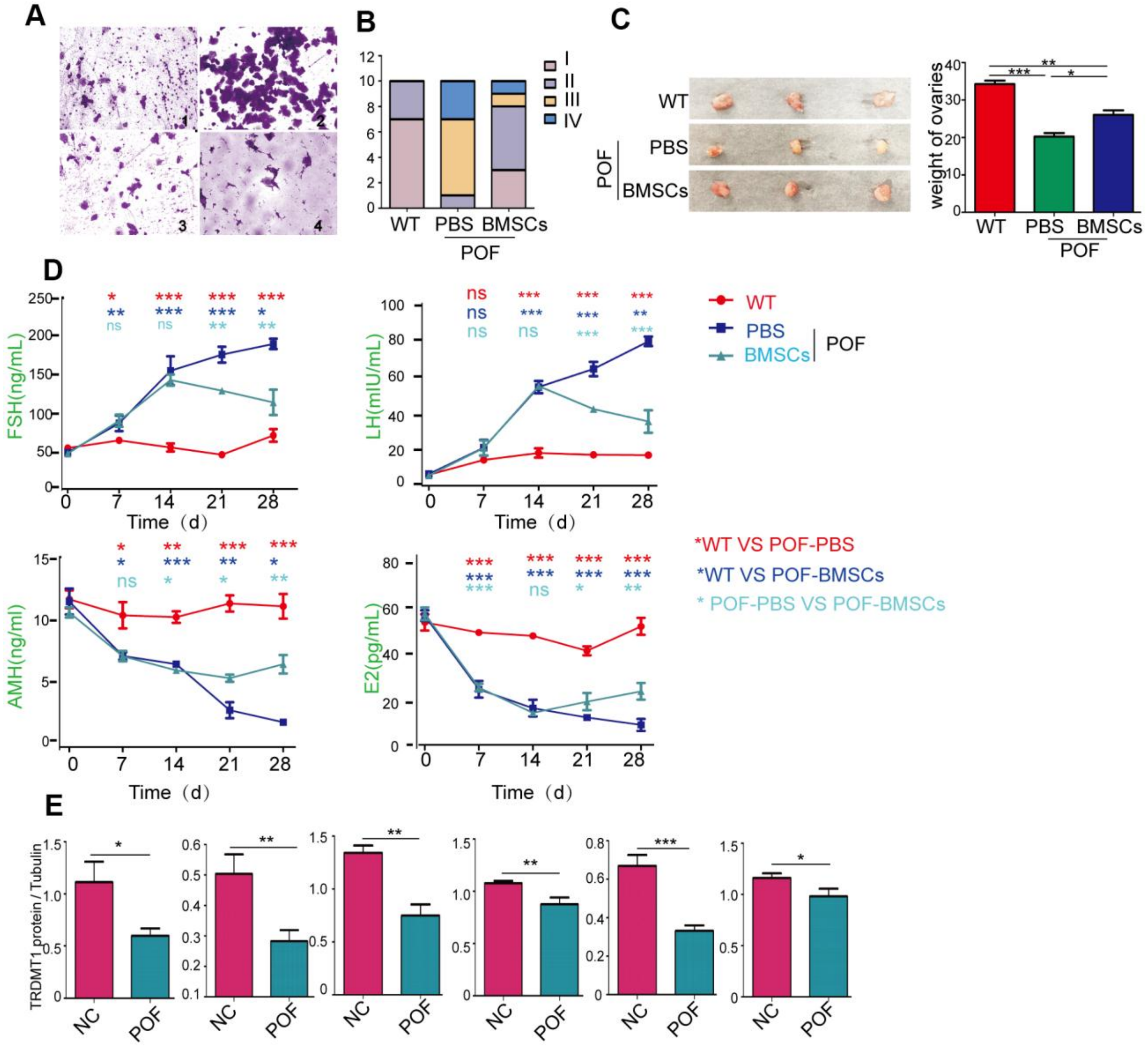

Supplementary Figure 1. (**A**) Normal estrous cycles: 1, proestrus; 2, estrus; 3, metestrus; and 4, diestrus. Scale bar: 100 µm. (**B**) Total numbers of rats from each group categorized into the various estrous patterns (I–IV). I, normal; II, regular cycles with shortened estrus; III, irregular cycles with prolonged diestrus and normal or prolonged estrus; and IV, no cyclicity. Total numbers of rats from each group categorized into the various estrous patterns (I–IV). (**C**) The weights of ovaries in the WT, POF-PBS and POF-BMSCs groups were measured. (**D**) Trends for the average levels of FSH, LH, AMH and E2. Comparison of the AMH, E2, FSH, and LH levels between groups at different times. (**E**) Quantification of Western blot data. The "ns" ($P > 0.05$) indicates no statistically significant difference. *$P < 0.05$, **$P < 0.01$, and ***$P < 0.001$ for all figures. Statistical significance was determined using two-tailed t-tests for two groups and ANOVA for multiple comparisons. All values are means ± SD.

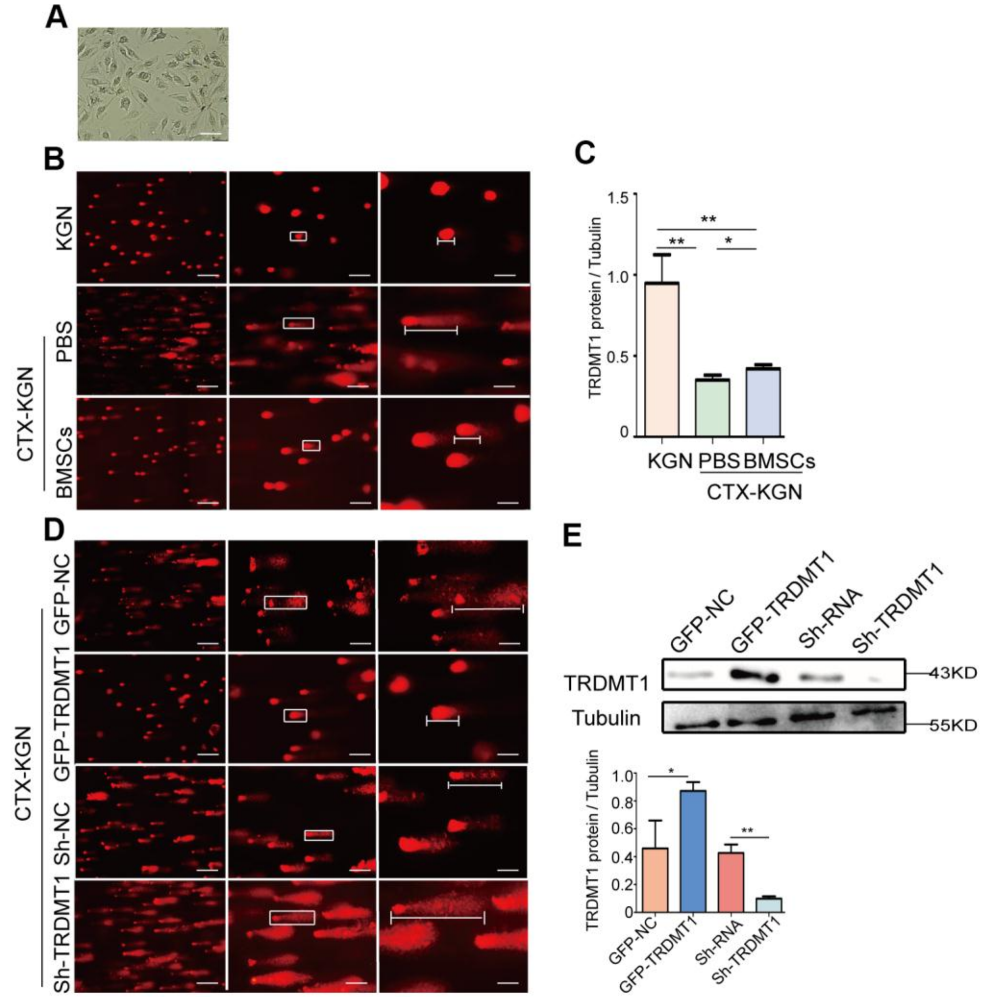

Supplementary Figure 2. (**A**) Morphology of GCs. Scale bar: 100μm. (**B**, **D**) Results of comet assay with different magnifications. (**C**) Quantification of Western blot data. (**C**) Quantification of Western blot data. (**E**) CTX-KGN transfected with GFP-NC, GFP-TRDMT1, Sh-RNA and Sh-TRDMT1 were confirmed by Western blotting.

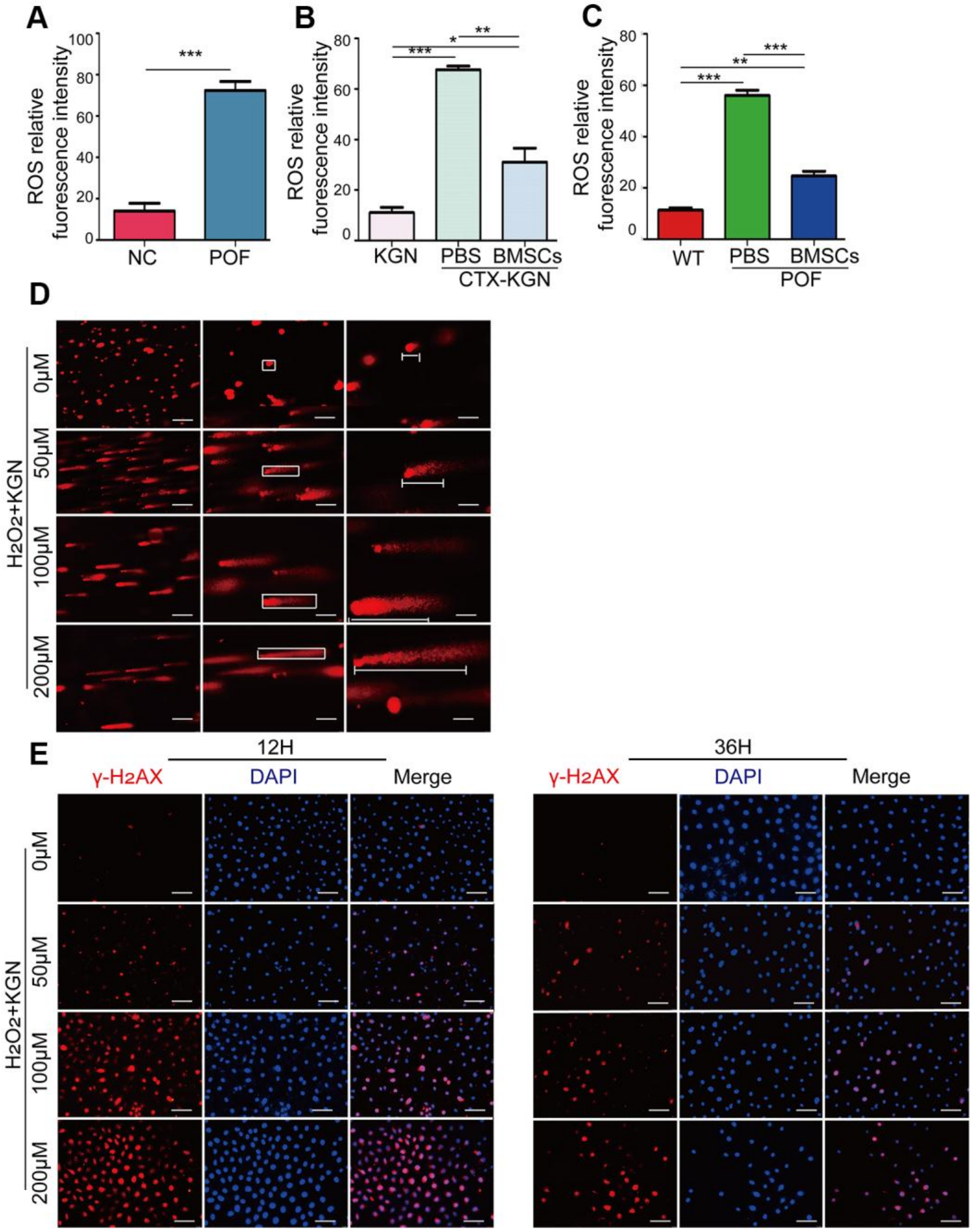

Supplementary Figure 3. (**A**) ROS relative fluorescence intensity was measured in NC and POF groups (**B**) ROS relative fluorescence intensity was measured in KGN, CTX-KGN-PBS and CTX-KGN-BMSCs groups. (**C**) ROS relative fluorescence intensity was measured in the ovaries of the WT, POF-PBS and POF-BMSCs groups. (**D**) Results of comet assay with different magnifications. (**E**) A wider view with multiple cells for γ-H_2AX immunofluorescence images. Scale bar:100µm *P<0.05, **p<0.01, and ***p<0.001. Statistical significance was determined using two-tailed t-tests for two groups and ANOVA for multiple comparisons. All values are means ± SD.

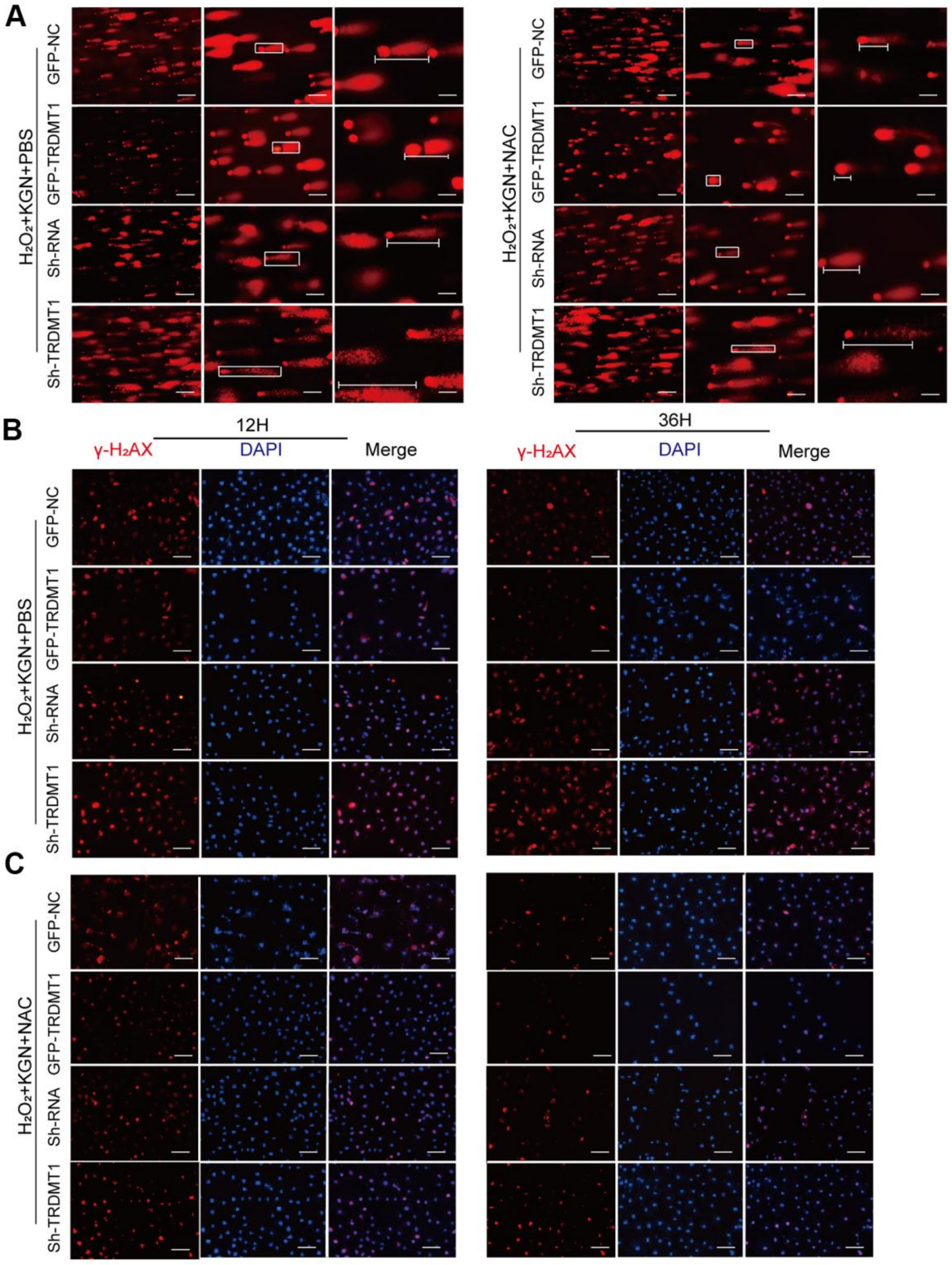

Supplementary Figure 4. (**A**) Results of comet assay with different magnifications. (**B**, **C**) A wider view with multiple cells for γ-H_2AX immunofluorescence images. Scale bar:100µm.

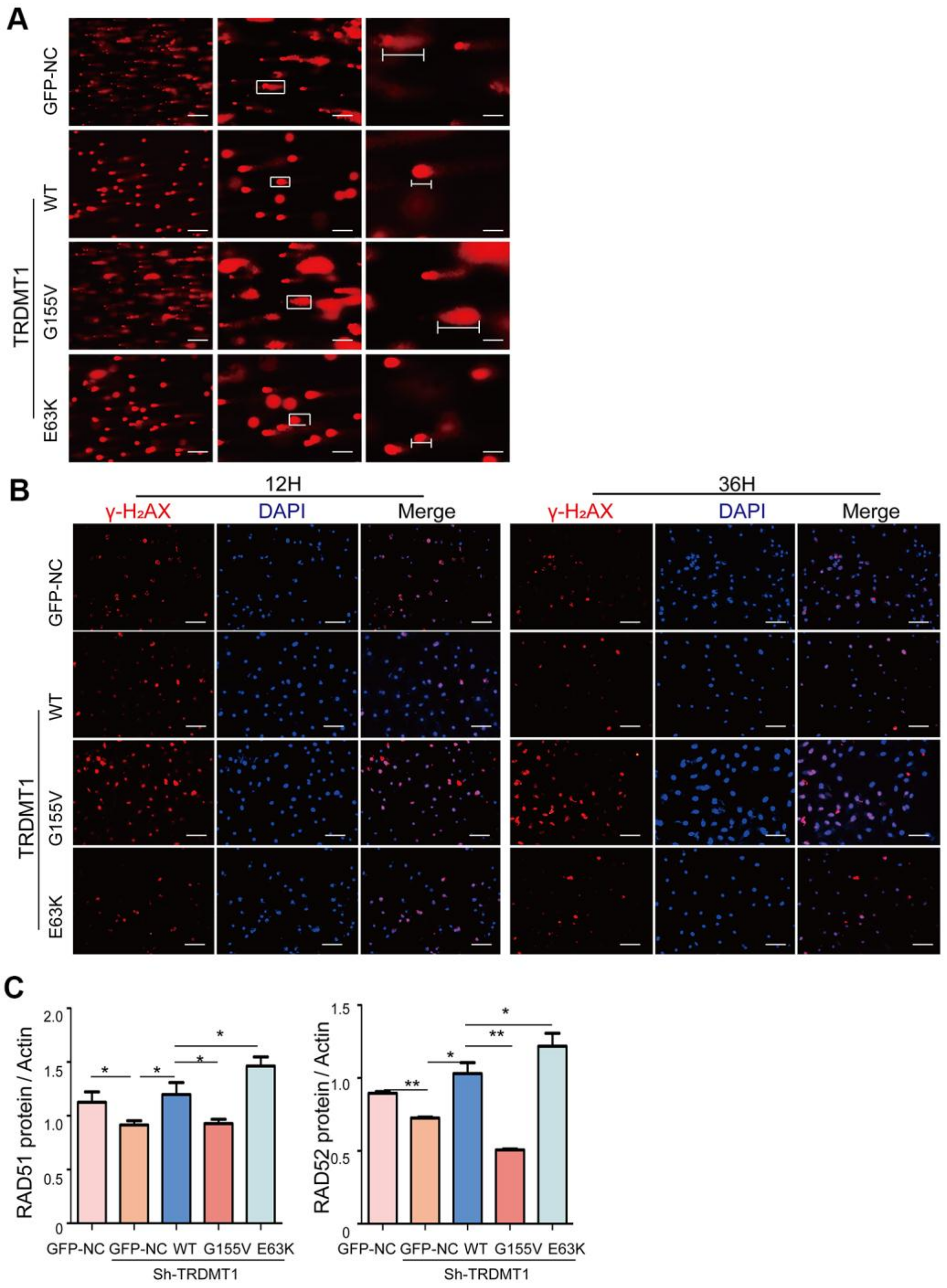

Supplementary Figure 5. (**A**) Results of comet assay with different magnifications. (**B**) A wider view with multiple cells for γ-H_2AX immunofluorescence images. Scale bar: 100μm. (**C**) Quantification of Western blot data.
